# HIV and hepatitis C risk among Tajik migrant workers who inject drugs in Moscow

**DOI:** 10.21203/rs.3.rs-2622346/v1

**Published:** 2023-02-27

**Authors:** Mary Ellen Mackesy-Amiti, Judith A. Levy, Mahbatsho Bahromov, Jonbek Jonbekov, Casey M. Luc

**Affiliations:** University of Illinois at Chicago; University of Illinois at Chicago; PRISMA Research Center; PRISMA Research Center; University of Illinois at Chicago

**Keywords:** HIV, hepatitis C, risk behavior, injection drug use, peer networks, Tajik migrant worker

## Abstract

**Background.:**

The HIV epidemic in Eastern Europe and Central Asia continues to grow with most infections occurring in high-risk groups including people who inject drugs and their sexual partners. Labor migrants from this region who inject drugs while in Russia are at especially high HIV risk.

**Methods.:**

We recruited 420 male Tajik migrant workers who inject drugs in Moscow for a peer-education HIV prevention intervention trial. Participants were interviewed about their sex and drug use behavior and tested for HIV and hepatitis C prior to the intervention.

**Results.:**

Over half of the men reported injecting with a previously used syringe in the past month. Many men reported condomless sex (42%), multiple sex partners (30%), and sex with sex workers (42%). Only 17% had ever been tested for HIV. Despite substantial risk behavior, prevalence rates of HIV (6.8%) and HCV (2.9%) although elevated were lower than expected when compared to estimates of prevalence among PWID at the national level in Tajikistan. Risk behavior in diaspora varied across the men’s regional area of origin in Tajikistan and occupation in Moscow with HIV prevalence rates highest among those working at the bazaars.

**Conclusion.:**

Tajik male migrants who inject drugs in Moscow are at heightened risk for HIV and hepatitis C. Evidence-based prevention approaches and messaging that specifically address the drug- and sex-related risk behavior of migrants from different parts of Tajikistan, employment sectors within the destination city, and socio-demographic background are needed.

## Introduction

Significant progress has been made in addressing the AIDS pandemic throughout much of the world with new infections declining globally by 32% from 2010 to 2021 (1). In Eastern Europe and Central Asia (EECA), however, new infections increased by 48% in the same time period (2). UNAIDS reported that in 2015, more than 80% of the region’s new infections were in the Russian Federation (3). Labor migration is a major contributor to the movement and exchange of HIV across country borders and populations including the EECA (4–6). In Russia, many migrant workers originate from the Central Asian countries, including Tajikistan - a small country with comparatively lower HIV rates, high poverty, and an ongoing opioid epidemic (7–9).

Although the HIV epidemic in the Russian Federation has generalized throughout its population (10), injection drug use remains an important risk factor accounting for about 40% of new infections in recent years (2, 11). While UNAIDS estimates that 7.2% of people who inject drugs (PWID) in the Eastern European region are living with HIV (12), Russian sources estimate that 28% of PWID in Russia are living with the virus (13). Labor migrants who inject drugs while in Russia are at especially high risk for acquiring HIV due to social marginalization and lack of access to health care and prevention services (14). Meanwhile, migrants who become infected with HIV while living in the Russian Federation are at risk of deportation. They also face a difficult choice between losing their income by returning home for treatment or remaining in Russia without access to HIV medical care (15).

To address the need for preventive interventions for this population, we developed and pilot tested the Migrants’ Approached Self-Learning Intervention in HIV/AIDS for Tajiks (MASLIHAT) intervention model for reducing risky drug, alcohol, and sexual behavior among male Tajik migrants who inject drugs while living in Moscow. MASLIHAT is a network-based, peer educator training intervention developed as a socio-cultural adaption of the Self-Help in Eliminating Life-Threatening Diseases (SHIELD) model (16). Pilot testing conducted in 2018 demonstrated promising results with significant declines in risk behavior over six months among both peer educator participants (PEs) and their network members with whom they regularly interacted (17).

We are currently conducting a cluster-randomized parallel groups trial to test the efficacy of the MASLIHAT intervention versus a comparison condition. This paper reports the risk behavior and HIV/HCV prevalence rates obtained from the study’s baseline survey and biological testing conducted with the combined sample of PEs and their network members recruited for both arms of the trial prior to the intervention. Our analysis contributes to a slim but highly concerning literature documenting HIV risk and acquisition among labor migrants in diaspora. While most labor migrant studies tend to concentrate primarily on sexual risk (6, 18–23), our data address a significant gap in what is known about migrants who engage in high-risk drug injection behavior while away from their home country. Our focus on HIV risk in Russia is highly relevant as the country is the major destination for labor migrants from the Central Asian countries of Tajikistan, Kyrgyzstan, Uzbekistan, and in recently lessening numbers from Kazakhstan (24, 25). Attempts to curb HIV among PWID globally will never succeed without understanding and subsequently addressing the factors that drive transmission among Central Asia’s people who inject drugs (PWID) at home and in other countries (26).

## Methods

Study procedures were reviewed and approved by the Institutional Review Boards of the University of Illinois Chicago, PRISMA Research Center in Tajikistan, and the Moscow Nongovernment Organization “Bridge to the future.”

### Recruitment.

From October 2021 to April 2022, we recruited and trained 140 male Tajik migrant workers as peer educators from 12 sites in Moscow: 2 Tajik diaspora organizations, 4 bazaars, and 6 construction work sites. To be eligible to participate as a PE assigned to either the MASLIHAT intervention or the comparison control condition (Healthy Living), prospective participants needed to be a male Tajik migrant aged 18 or older, a current or former PWID, give informed consent, intending to reside in Moscow for the next 12 months to participate in their assigned intervention and follow-up data collection, and willing to recruit two male PWID to participate as IDU network members for baseline and follow-up interviewing but who would not participate in the MASLIHAT or control condition educational sessions or activities. Network members (n=280) had to meet the same eligibility criteria as PEs but also: 1) have injected drugs at least once in the last 30 days; and 2) be someone whom the PE sees at least once a week to permit them to share intervention information and encourage possible normative and behavioral change within their social networks. Participants in both study conditions received the customary compensation in Moscow of $20.00 for their time and transportation costs in participating in intervention sessions (PEs only) and for being interviewed at baseline and follow-up (both PEs and network members).

### Baseline interviews.

After giving informed consent, baseline interviews with PEs and network members were conducted at the PRISMA office in Moscow or at a private location of the participant’s choosing. Following the interview, participants were referred to the Moscow HIV Prevention Center to be tested for HIV and hepatitis C virus (HCV). Anonymized test results were reported to study staff with only a group number to identify the recruitment site.

### Measures.

The structured baseline questionnaire collected information on sociodemographic characteristics, migration characteristics and community involvement, substance use prior to migration and in the past six months in Moscow, sexual risk behavior, PWID network and injection risk behavior, HIV-related knowledge and attitudes, and psychosocial measures including depression, loneliness, and drug use and migrant stigma.

#### HIV knowledge and risk a wareness

HIV knowledge was assessed with 13 items (Cronbach alpha = 0.91). Eight items assessed knowledge of HIV transmission and non-transmission routes with response options being “safe,” “unsafe,” or “not sure.” Five additional true-false items assessed HIV-related knowledge such as, “You can look at a person and tell if they are infected with HIV,” and “There is a cure for HIV.” Correct responses were summed for a possible total score of 13 with responses of “not sure” counted as incorrect. Participants also were asked to rate their likelihood of contracting HIV as “not at all likely,” “somewhat likely,” or “very likely.”

#### Alcohol use

Alcohol use measures included frequency of alcohol use in the past month and the AUDIT assessment of hazardous alcohol use and alcohol dependence (27). Participants were asked, “How many days in the past month have you used alcohol, including beer, wine, or vodka?” Heavy drinking was assessed with AUDIT question #3, “How often do you have 6 or more drinks on one occasion,” with responses on a 5-point scale from “never” to “daily or nearly daily.” These responses were dichotomized for analysis as “never or less than monthly” vs. “at least monthly.”

#### Sexual risk behavior

Sexual risk behavior outcomes included having sex with a sex worker, multiple sex partners, and having sex without a condom in the past month. Past month condom use with regular and casual sexual partners and with sex workers was assessed separately on a 4-point scale as “never,” “sometimes,” “often,” or “always.” The items were combined to create a binary measure of “any condomless sex” vs. “sex with condoms or no sexual activity” in the past month.

#### Injection risk behavior.

Recent syringe sharing was assessed in response to the question, “When was the last time that you used a needle to shoot drugs after someone else used it first?” with 6 options: never, more than 4 weeks ago, in the last 4 weeks, within the last week, yesterday, or today. Responses were dichotomized to create a binary measure of having or not having used a shared syringe within the past month. Participants also were asked about syringe cleaning practices with the question, “When you have used a needle after someone else, how often did you clean the syringe with [bleach, alcohol, water only, soap and water, or nothing]” with response options on a 6-point scale from “never” to “always.”

#### Area of residence in Tajikistan.

Participants were asked to select from a list the area of Tajikistan in which they had resided before migrating to Moscow. The list contained Tajikistan’s 4 administrative regions (the provinces of Khatlon, Sughd, and Gorno-Badakhshan Autonomous Oblast (GBAO), and the Districts of Republican Subordination - in Russian referred to as Rayoni Respublicanskogo Podchinenie (RRP)), and Dushanbe (Tajikistan’s capital city).

### Analysis.

We compared the sociodemographic attributes of PEs with those of their network members to identify possible significant differences using linear regression and multinomial logit models with cluster-adjusted standard errors. We conducted bivariate tests of association to explore the relationships between 3 types of risk behavior (regular vs. heavy alcohol use, syringe sharing in the past month, and condomless sex in the past month) and the sociodemographic characteristics of participants using population-averaged Poisson regression models with an exchangeable within-group correlation structure and robust standard error estimation to obtain risk ratios and 95% confidence intervals.

## Results

Table 1 shows the baseline demographic characteristics of PEs (N=140) and network members (N=280). PEs and network members reported similar demographic profiles. Network members were on average one year younger than PEs (B = −1.07, 95% CI −1.69 - −0.46) and more likely to be on their first trip (Wald χ^2^ = 7.49, p = .02).

Of the 420 participants, only 17% (n=71) had been tested for HIV prior to participating in the study with one reporting that he tested positive and 14 declining to disclose their results. Participants recruited through a diaspora organization were more likely to have been tested (31 %) compared to those recruited from construction sites (18%) or bazaars (8%). When requested to be tested for the study, 7 declined (1.7%). Of the 413 tested, 28 (6.8%) were HIV positive and 12 (2.9%) were HCV positive. All 12 who tested positive for HCV also tested positive for HIV. Participants recruited from the bazaar sites were more likely than participants from construction sites to test positive for HIV (13.3% vs. 5.2%) and HCV (7.1 % *vs*. 1.4%). None of the participants recruited from the diaspora sites tested positive for either HIV or HCV. HIV knowledge and awareness responses are shown in Table 2. The average HIV transmission knowledge score was 7.2 (SD 3.3), or 55% correct. Migrants employed in loading and food service in the bazaars had lower knowledge scores compared to those employed in construction (Wald χ^2^=17.33, p=0.0017), while those who had tested previously for HIV irrespective of occupation had higher knowledge scores (b = 2.07, 95% CI 1.24–2.89). More than one-third of participants who reported they had tested HIV negative prior to enrollment in the study believed they had no risk of acquiring HIV and were not at all worried, while less than 10% felt their risk was high and worried a lot.

Prevalence of high-risk alcohol use, sexual risk behaviors, and injection risk behaviors are presented in Table 3. A large proportion of the men (39%) drank alcohol more than once a week, and 21 % reported heavy drinking (6 or more drinks at a time) at least once a month. Sexual risk behavior was common, with 42% reporting condomless sex in the past month. Over 50% reported injecting with a previously used syringe in the past month. Among these, few (7%) ever cleaned their syringes with bleach and 58% never cleaned their syringes with anything.

Associations between risk behaviors and sociodemographic characteristics (risk ratios and 95% confidence intervals) are shown in Table 4. Most participants had traveled to Moscow for work at least once previously, and the typical length of stay so far for this trip was 2–3 years. Migrants who came from the Dushanbe area had the lowest rates of heavy alcohol use and syringe sharing while those from the Gorno-Badakshan province (GBAO) had the highest rates. Migrants who had attended university but left without a degree and those who were divorced were more likely to drink heavily. Condomless sex was most prevalent among migrants from the RRP and least prevalent among those from the Sughd province. Men with a college or university degree and those who were married or previously married were less likely on average to engage in condomless sex. Education and marital status had no bearing on the likelihood of using shared syringes. Risk behavior varied by employment category with men employed in construction less likely to engage in heavy alcohol use but more likely to report syringe sharing compared to their counterparts employed in selling and food service in the bazaars.

## Discussion

Tajikistan faces numerous challenges in calculating how close it has come to meeting its UNAIDS Fast Track target - Ending the AIDS Epidemic by 2030. Almost one million Tajik citizens work outside the country at any given time with Russia the major destination. Such high rates of migration make it difficult to estimate the total number of Tajiks in-country and throughout the world who are at risk for and living with HIV (28). Our findings on Tajik male migrants who inject drugs while living in Moscow help to address the need for epidemiological data on HIV/HCV risk and prevalence among Tajik migrants in diaspora.

Our findings confirm that Tajik labor migrants who inject drugs are at considerable risk for HIV/HCV when living in Moscow, a high HIV/HCV prevalence destination city. Most participants in our study reported using a shared syringe without first cleaning it, and a large proportion reported condomless sex, multiple sex partners, and sex with sex workers. Many also engaged in heavy alcohol use on a regular basis, a lifestyle practice that can encourage risky drug and sexual behavior (29).

Due to COVID travel restrictions, most of the participants in the current study had been in Moscow for 2 years or more; less than 10% had been in Moscow less than one year as compared with the over one third of our earlier pilot study sample (17). Also, participants in the current study were more likely to report being at least somewhat worried about HIV (59% vs. 40%). Perhaps the advent of COVID during the interim since the pilot was conducted has increased public general awareness, even among Tajik migrants (24), of being personally vulnerable to serious illness, loss of work, and other adverse consequences as the result of all types of viral transmission including HIV and HCV.

Our findings that report the influence of demographic characteristics on sex and alcohol risk suggest that marital status and education tend to exert a positive effect on migrant risk behavior. Yet education and marital status appear to have no bearing on the likelihood of sharing an uncleaned syringe. Possibly, irrespective of a participant’s demographic background, sharing a syringe, even if unclean, is the result of most migrants’ fear of being reported to authorities as a PWID when obtaining new or clean syringes from a local pharmacy or needle exchange.

HIV prevalence rates were significantly higher among bazaar workers than those working in construction, while no cases were found among migrants recruited from the diaspora organizations. These differing results may be related to prior testing exposure. Participants recruited from diaspora organizations and those employed in construction were more likely to have been among the 17% in our study who had previously tested for HIV. Meanwhile prior testing was associated with greater knowledge of HIV risk and prevention, which also may help to explain why these two recruitment groups exhibit lower prevalence rates.

Area of origin in Tajikistan appears to strongly influence participants’ HIV/HCV risk behavior while in Moscow. Each of these areas embody a somewhat different economic, cultural, and geographical environment that, in turn, may contribute to the differences found in HIV/HCV risk and prevention behavior among migrants who originate from them. Unlike the urbanized and comparatively well-resourced city of Dushanbe, Gorno-Badakshan (GBAO) is the poorest and most remote area of the country (30). With no major industries its unemployment rates are exceedingly high, and its small population differs ethnically and linguistically from the rest of Tajikistan. Migrants from GBAO tend to stay longer at their destination point and return home less frequently due to the remoteness of the area. Staying in Russia for long periods of time without citizenship, these migrants have little access to health care and information. When compared with each other, such regional socio-economic and geographical differences may help in some way to explain why participants from Dushanbe reported the lowest rates of heavy alcohol use and syringe sharing while those who migrated from the GBAO province reported the highest. Data from the Tajikistan Ministry of Health surveillance system (2006–2019) also shows that among people living with HIV, migrants (compared to non-migrants) were more likely to be from GBAO (31). Meanwhile, condomless sex was the most prevalent among migrants from the RRP and the least prevalent among those from the Sughd province. Sughd is the second largest province in Tajikistan and is the most industrialized and economically developed. With special economic development zones and good employment opportunities, this region has better health care provision, including greater access to HIV counseling and testing facilities.

Of the 420 men who participated in our study, only 17% (n=71) had been tested for HIV prior to participating in the study. Such a low testing rate is cause for concern. Of the 413 men who agreed to being tested at the study’s baseline, 28 (6.8%) tested positive for HIV and 12 (2.9%) tested positive for HCV. Labor migrants in diaspora avoid HIV testing out of fear of being deported, losing their current employment, or encountering stigma and social discrimination if diagnosed as HIV positive. Migrants who are unknowingly HIV positive lack the HIV treatment that they need until advancing symptoms drive them to seek medical attention. An analysis of data from the Tajikistan Ministry of Health surveillance system found that among migrants living with HIV, more time spent in Russia predicted late presentation (31). Such late presentation in initiating antiretroviral therapy can result in higher mortality and morbidity for migrants than might have occurred if begun sooner (32) and hinders government efforts in low resource countries to reaching the global fast-track goal of 90% of people living with HIV receiving treatment (33). In addition, migrants who become unknowingly infected in a destination country can unintentionally transmit the virus to their spouses and other sexual partners upon returning home (34).

Despite overall high levels of risk behavior and low testing rates, HIV and HCV prevalence (6.8% HIV positive and 2.9% HCV positive) were relatively low among our study’s male PWID participants, compared to the estimated prevalence of HIV/HCV at the national level among PWID in Tajikistan (13.5% HIV and 24.9% HCV in 2011) (9) and in the Russian Federation (30% HIV, 69% HCV based on multiple estimates from 2009–2015) (35). The disparity may be at least partly due to sex differences, as national estimates include both men and women. In many settings women who inject drugs have a higher prevalence of HIV (36, 37). Also, the higher HIV prevalence rate for PWID in Tajikistan possibly reflects a sizable number of male Tajik migrants who return home for HIV treatment after acquiring the virus and/or testing positive in a destination country including Russia.

### Limitations.

The study’s participants were sampled through in-person recruitment at 10 occupational sites and by referral from 2 Tajik service networks. Consequently, the study’s results may not generalize to those migrants whom these methods failed to reach or who chose not to participate. Also, biological testing for HIV and HCV was conducted by the Moscow HIV Prevention Center, an organization separate from the study that reported the results without personal identifiers to the MASLIHAT research staff. This anonymity was necessitated by the migrants’ reluctance to agree to confidential testing if their results could be linked to their identity and personal information. This safeguard, however, hinders our ability to test for possible associations between a participant’s HIV status and personal characteristics such as HIV knowledge, Tajikistan area of origin, and risk behavior. Also, our sample consists solely of men. Reliable statistics on the number of Tajik women who migrate to Russia for work are not readily available, although known to be low when compared to men. Nonetheless, their growing numbers (38) suggest the need for research that also considers their HIV/HCV risk behavior and prevalence while in diaspora. Areas of foci include whether or not culturally-defined gender expectations, drug use, and employment in different economic sectors of the Russian Federation translate to differences in female risk behavior. Finally, our data are cross-sectional. We will have to wait until the study’s longitudinal data collected at three follow-up time points are available to examine possible causal pathways.

## Conclusions

The alarming rate of HIV/HCV risk behavior among Tajik migrants who inject drugs in Moscow, and likely in other destination cities throughout Russia, call for heightened prevention strategies and programs designed to reach this vulnerable population. Tajik migrants who inject drugs are not homogenous, however, in their HIV/HCV risk behavior. Evidence-based prevention approaches and messaging that specifically address the drug- and sex-related risk behavior of migrants from different parts of Tajikistan, employment sectors within the destination city, and socio-demographic background are needed.

## Figures and Tables

**Table 1:** Demographic characteristics of PWID enrolled in the MASLIHAT trial

	Peer educator (n=140)*		Network members (n=280)*		
Variable	*Mean (SD)*	*Range*	*Mean (SD)*	*Range*	*P-value* ^†^
Age	30.7	21–50	29.6	19–49	0.001
		(6.74)		(5.90)	
	***n***	***%***	***n***	***%***	
Recruitment site					
Diaspora organization	24	17.1	48	17.1	
Bazaar	44	31.4	88	31.4	
Construction site	72	51.4	144	51.4	
Area of origin					0.280
Dushanbe	31	22.1	57	20.4	
Khatlon	29	20.7	59	21.1	
Sughd	13	9.3	31	11.1	
Gorno-Badakhshan	54	38.6	105	37.5	
Subordinate Districts	13	9.3	28	10.0	
Education					0.374
Secondary or less	91	65.0	165	58.9	
College or technical college	32	22.9	73	26.1	
University but no degree	6	4.3	8	2.9	
University degree	11	7.9	34	12.1	
Marital Status					0.200
not married	65	46.4	111	39.9	
married	14	10.0	38	13.7	
divorced	61	43.6	129	46.4	
How long in Russia this trip					0.280
One year or less	9	6.6	30	10.9	
> 1 to 2 years	42	30.7	88	32.1	
> 2 years	86	62.8	156	56.9	
How many trips to Moscow					0.024
One	8	5.7	41	14.6	
Two	55	39.3	81	28.9	
Three or more	77	55.0	158	56.4	
Employment					0.885
Construction	76	54.3	154	55.0	
Loading in bazaar	29	21.7	58	20.7	
Selling/food service	27	19.3	48	17.1	
Other / Missing	8	5.7	20	7.1	

* Study characteristics may not sum to column N due to missingness

† Linear regression for continuous and multinomial logit models for categorical variables with cluster-adjusted standard errors

**Table 2.** HIV knowledge and awareness among PWID enrolled in the MASLIHAT trial (N=420)

Variable	Mean	IQR
HIV knowledge score	7.21	5 – 10
	**n**	**%**
How likely to get HIV		
Not at all Likely	149	36.3
Somewhat Unlikely	121	29.4
Somewhat Likely	107	26.0
Very Likely	34	8.3
How likely at least one person you inject drugs with has HIV		
Not at all Likely	150	36.5
Somewhat Unlikely	146	35.5
Somewhat Likely	85	20.7
Very Likely	30	7.3
How likely you have shared syringes with someone who has HIV		
Not at all Likely	157	38.4
Somewhat Unlikely	138	33.7
Somewhat Likely	86	21.0
Very Likely	28	6.9
How much you worry about HIV		
Not at all	170	40.6
Somewhat	217	51.8
A Lot	32	7.6

Note: Study characteristics may not sum to Total N due to missingness

**Table 3.** Risk behavior among PWID enrolled in the MASLIHAT trial (N=420)

Variable	n	%
Frequency of alcohol use		
Monthly or less	70	16.7
2 to 4 times a month	187	44.6
2 to 3 times a week	142	33.9
4 or more times a week	20	4.8
Heavy alcohol use		
Never	218	52.2
Less than monthly	112	26.8
Monthly	72	17.2
Weekly	13	3.1
Daily or Nearly Everyday	3	0.7
Sex with sex worker past month	177	42.1
Multiple sex partners past month	125	29.8
Any condomless sex past month	178	42.4
Injected with shared syringe past month	228	54.3
Frequency syringe sharing past 3 months		
Never	101	24.1
Rarely	112	26.7
Less than half or half the time	90	21.4
About Half the Time	70	16.7
More than half the time	34	8.1
Almost Always	11	2.6
Always	2	0.5
Cleaned syringe with bleach*		
Never	380	90.7
Once or twice / a few times	37	8.8
Sometimes / often	2	0.5
Cleaned syringe with water		
Never	265	63.2
Once or twice / a few times	131	31.3
Sometimes / often	23	5.5

Note: Study characteristics may not sum to Total N due to missingness

**Table 4.** Associations between risk behaviors and sociodemographic characteristics of PWID enrolled in the MASLIHAT trial

Variable	*Heavy alcohol use (N=418)*		*Syringe Sharing (N=420)*		*Condomless Sex (N=420)*	
	*PR*	*95% Conf. Int*.	*PR*	*95% Conf. Int*.	*PR*	*95% Conf. Int*.
Age	1.02	(0.99, 1.05)	1.00	(0.98, 1.02)	0.96	(0.93, 0.98)
Recruitment site						
Diaspora organization	1.00		1.00		1.00	
Bazaar	0.98	(0.57, 1.67)	1.57	(0.99, 2.50)	0.83	(0.57, 1.20)
Construction site	0.71	(0.43, 1.15)	1.75	(1.12, 2.72)	0.86	(0.61, 1.21)
Area of Origin						
Dushanbe	1.00		1.00		1.00	
Khatlon	1.31	(0.66, 2.59)	1.48	(1.04, 2.10)	0.91	(0.61, 1.35)
Sughd	1.50	(0.69, 3.28)	1.54	(1.11, 2.15)	0.47	(0.28, 0.79)
Gorno-Badakhshan	2.06	(1.22, 3.49)	1.69	(1.25, 2.28)	1.23	(0.90, 1.67)
RRP	_-_^†^		1.23	(0.77, 1.97)	1.82	(1.30, 2.53)
Education						
Secondary or less	1.00		1.00		1.00	
College or technical college	1.41	(0.94, 2.11)	0.93	(0.78, 1.11)	0.53	(0.38, 0.73)
University but no degree	2.38	(1.18, 4.79)	1.00	(0.61, 1.62)	0.55	(0.24, 1.26)
University degree	1.10	(0.58, 2.07)	0.99	(0.78, 1.27)	0.38	(0.20, 0.74)
Marital Status^a^						
not married	1.00		1.00		1.00	
married	0.66	(0.32, 1.38)	1.25	(0.96, 1.63)	0.32	(0.18, 0.57)
divorced	1.60	(1.09, 2.33)	1.04	(0.83, 1.31)	0.55	(0.44, 0.69)
How many trips to Moscow						
one	1.00		1.00		1.00	
two	1.46	(0.72, 2.98)	0.76	(0.58, 1.01)	1.08	(0.76, 1.54)
three or more Employment	1.59	(0.74, 3.41)	0.83	(0.64, 1.07)	0.72	(0.51, 1.04)
Construction	1.00		1.00		1.00	
Loading in bazaar	1.12	(0.72, 1.74)	0.81	(0.60, 1.10)	1.20	(0.94, 1.54)
Selling/food service	1.79	(1.18, 1.74)	0.63	(0.44, 0.91)	0.72	(0.51, 1.01)
Other / missing	0.40	(0.12, 1.38)	1.31	(1.08, 1.58)	0.81	(0.44, 1.50)

a 2 missing

† Excluded due to non-varying outcome, N=377

RRP: Region of Republican Subordination

## Data Availability

The datasets supporting the conclusions of this article are available in the Open Science Framework repository [Project ID: osf.io/7g3yh Data link: https://osf.io/ws5mp/]

## Abbreviations

EECA: Eastern Europe and Central Asia
GBAO: Gorno-Badakhshan Autonomous Oblast
HCV: Hepatitis C Virus
HIV: Human Immunodeficiency Virus
MASLIHAT: Migrants’ Approached Self-Learning Intervention in HIV/AIDS for Tajiks
PE: Peer educator
PWID: People who inject drugs
RRP: Rayoni Respublicanskogo Podchinenie (Region of Republican Subordination)

## References

[1] UNAIDS. Global HIV & AIDS statistics - 2022 Fact Sheet [Internet]. 2022. Accessed 1 Feb 2023. Available from: http://www.unaids.org/en/resources/fact-sheet.

[2] UNAIDS. In Danger: UNAIDS Global AIDS Update 2022. Geneva: Joint United Nations Programme on HIV/AIDS; July 27, 2022. Available from: https://www.unaids.org/en/resources/documents/2022/in-danger-global-aids-update

[3] UNAIDS. Prevention Gap Report. Geneva: Joint United Nations Programme on HIV/AIDS; July 11, 2016. Available from: http://www.unaids.org/en/resources/documents/2016/prevention-gap

[4] UNAIDS, International Organization for Migration. Migration and AIDS. International Migration. 1998;36(4):445–68. 12295093

[5] Del Amo J, Broring G, Hamers FF, Infuso A, Fenton K. Monitoring HIV/AIDS in Europe’s migrant communities and ethnic minorities. AIDS. 2004;18(14):1867–73. 15353972 10.1097/00002030-200409240-00002

[6] El-Bassel N, Gilbert L, Shaw SA, Mergenova G, Terlikbayeva A, Primbetova S, The Silk Road Health Project: How mobility and migration status influence HIV risks among male migrant workers in Central Asia. PLOS ONE. 2016;11(3):e0151278. 26967159 10.1371/journal.pone.0151278 PMC4788424

[7] World Health Organization (WHO). Epidemiological Factsheet: Tajikistan. UNAIDS: Joint UN Programme on HIV/AIDS; 2010. Available from: http://aidsinfo.unaids.org

[8] United Nations Office on Drugs and Crime. Opiate Flows through Northern Afghanistan and Centre Asia: A Threat Assessment. Geneva: UNODC; May 2012. Available from: https://www.unodc.org/documents/data-and-analysis/Studies/Afghanistan_northern_route_2012_web.pdf

[9] Zabransky T, Mravcik V, Talu A, Jasaitis E. Post-Soviet Central Asia: A summary of the drug situation. Int J Drug Policy. 2014;25(6):1186–94. 24954816 10.1016/j.drugpo.2014.05.004

[10] Burchell AN, Calzavara LM, Orekhovsky V, Ladnaya NN. Characterization of an emerging heterosexual HIV epidemic in Russia. Sex Transm Dis. 2008;35(9):807–13. 18496470 10.1097/OLQ.0b013e3181728a9e

[11] Stuikyte R, Barbosa I, Kazatchkine M. Getting to grips with the HIV epidemic in Russia. Current Opinion in HIV and AIDS. 2019;14(5):381–6. 31261159 10.1097/COH.0000000000000573

[12] UNAIDS. UNAIDS Global AIDS Monitoring 2022. Available from: https://aidsinfo.unaids.org/.

[13] Federal Scientific and Methodological Center for the Prevention and Control of AIDS in Russia.

[14] Weine SM, Kashuba AB. Labor migration and HIV risk: A systematic review of the literature. AIDS Behav. 2012;16(6):1605–21. 22481273 10.1007/s10461-012-0183-4 PMC3780780

[15] UNAIDS. Listening to the voices of migrants living with HIV in the Russian Federation [Internet]. 15 Jun 2021. Accessed 1 Feb 2023. Available from: https://www.unaids.org/en/resources/presscentre/featurestories/2021/june/20210615_migrants-russian-federation.

[16] HIV/AIDS Prevention Research Synthesis Project. Compendium of Evidence-Based Interventions and Best Practices for HIV Prevention. Self-Help in Eliminating Life-Threatening Diseases (SHIELD): Centers for Disease Control and Prevention.

[17] Levy JA, Mackesy-Amiti ME, Bakhromov M, Jonbekov J, Latkin CA. A network-based HIV prevention intervention for Tajik migrant workers who inject drugs. AIDS Behav. 2022;26(3):719–27. 34387778 10.1007/s10461-021-03431-5 PMC8840980

[18] Zhang X, Rhoads N, Rangel MG, Hovell MF, Magis-Rodriguez C, Sipan CL, Understanding the impact of migration on HIV risk: An analysis of Mexican migrants’ sexual practices, partners, and contexts by migration phase. AIDS Behav. 2017;21(3):935–48. 27888370 10.1007/s10461-016-1622-4 PMC5837820

[19] Giorgio M, Townsend L, Zembe Y, Cheyip M, Guttmacher S, Carter R, HIV prevalence and risk factors among male foreign migrants in Cape Town, South Africa. AIDS Behav. 2017;21 (3):949–61. 27557987 10.1007/s10461-016-1521-8 PMC6004499

[20] Olawore O, Tobian AAR, Kagaayi J, Bazaale JM, Nantume B, Kigozi G, Migration and risk of HIV acquisition in Rakai, Uganda: a population-based cohort study. The Lancet HIV. 2018;5(4):e181–e9. 29490875 10.1016/S2352-3018(18)30009-2 PMC6195205

[21] Dias S, Gama A, Loos J, Roxo L, Simoes D, Nostlinger C. The role of mobility in sexual risk behaviour and HIV acquisition among sub-Saharan African migrants residing in two European cities. PLOS ONE. 2020;15(2):e0228584. 32023309 10.1371/journal.pone.0228584 PMC7001961

[22] Alessi EJ, Kahn S, Giwa S, Cheung S. ‘Those tablets, they are finding an empty stomach’: a qualitative investigation of HIV risk among sexual and gender minority migrants in Cape Town, South Africa. Ethn Health. 2022;27(4):800–16. 32894689 10.1080/13557858.2020.1817342

[23] Guerra-Ordoñez JA, Benavides-Torres RA, Zapata-Garibay R, Ruiz-Cerino JM, Ávila-Alpirez H, Salazar-Barajas ME. Percepción de riesgo para VIH y sexo seguro en migrantes de la frontera norte de México. Revista Internacional de Andrologia. 2022;20(2):80–5. 35078727 10.1016/j.androl.2020.10.010

[24] Poletaev DV. Life and health of labor migrants from Central Asia in Russia in the context of the COVID-19 pandemic. Population and Economics. 2022;6(4).

[25] Sharifzoda K. Why Is Kazakhstan a Growing Destination for Central Asian Migrant Workers? The Diplomat [Internet]. June 13, 2019. Accessed Feb 20, 2023. Available from: https://thediplomat.com/2019/06/why-is-kazakhstan-a-growing-destination-for-central-asian-migrant-workers.

[26] Deryabina AP El-Sadr WM. Optimizing HIV prevention and treatment outcomes for persons with substance use in Central Asia: what will it take? Current Opinion in HIV and AIDS. 2019;14(5). 10.1097/COH.0000000000000565 PMC6688715 31219889

[27] Saunders JB, Aasland OG, Babor TF, De La Fuente JR, Grant M. Development of the Alcohol Use Disorders Identification Test (AUDIT): WHO Collaborative Project on Early Detection of Persons with Harmful Alcohol Consumption-II. Addiction. 1993;88(6):791–804. 8329970 10.1111/j.1360-0443.1993.tb02093.x

[28] Pommersheim JM. How the U.S. Government and Tajikistan Partner to Fight HIV/AIDS [Internet]. 29 Nov 2019. Accessed 1 Feb 2023. Available from: https://tj.usembassy.gov/how-the-u-s-government-and-tajikistan-partner-to-fight-hiv-aids-2/.

[29] El-Bassel N, Marotta PL. Alcohol and sexual risk behaviors among male Central Asian labor migrants and non-migrants in Kazakhstan: Implications for HIV prevention. AIDS Behav. 2017;21 (2):183–92. 28983808 10.1007/s10461-017-1918-z PMC5859564

[30] Bromberg DJ, Tate MM, Alaei A, Rozanova J, Karimov S, Saidi D, “Who are you and what are you doing here?”: Social capital and barriers to movement along the HIV care cascade among Tajikistani migrants with HIV to Russia. AIDS Behav. 2021. 10.1007/s10461-021-03359-w PMC8903220 34195912

[31] Bromberg DJ, Tate MM, Alaei K, Karimov S, Saidi D, Alaei A. Association between time spent in the Russian Federation and late presentation for HIV among Tajikistani migrants. BMC Public Health. 2020;20(1):1379. 32912203 10.1186/s12889-020-09434-6 PMC7488340

[32] Guillon M, Celse M, Geoffard P-Y. Economic and public health consequences of delayed access to medical care for migrants living with HIV in France. The European Journal of Health Economics. 2018;19(3):327–40. 28343331 10.1007/s10198-017-0886-6

[33] Ingabire PM, Semitala F, Kamya MR, Nakanjako D. Delayed antiretroviral therapy (ART) initiation among hospitalized adults in a resource-limited settings: A challenge to the global target of ART for 90% of HIV-infected individuals. AIDS Res Treat. 2019;2019:1832152. 31057959 10.1155/2019/1832152 PMC6463639

[34] Golobof A, Weine S, Bahromov M, Luo J. The roles of labor migrants’ wives in HIV/AIDS risk and prevention in Tajikistan. AIDS Care. 2011 ;23(1):91–7. 21218281 10.1080/09540121.2010.498859 PMC3816794

[35] Degenhardt L, Peacock A, Colledge S, Leung J, Grebely J, Vickerman P Global prevalence of injecting drug use and sociodemographic characteristics and prevalence of HIV, HBV, and HCV in people who inject drugs: a multistage systematic review. The Lancet Global Health. 2017;5(12):e1192–e207. 29074409 10.1016/S2214-109X(17)30375-3 PMC5683738

[36] Des Jarlais DC, Feelemyer JP Modi SN, Arasteh K, Hagan H. Are females who inject drugs at higher risk for HIV infection than males who inject drugs: An international systematic review of high seroprevalence areas. Drug Alcohol Depend. 2012;124(1–2):95–107. 22257753 10.1016/j.drugalcdep.2011.12.020 PMC3353009

[37] UNAIDS. The Gap Report. Geneva: Joint United Nations Programme on HIV/AIDS (UNAIDS); 16 Jul 2014. Available from: https://www.unaids.org/en/resources/documents/2014/20140716_UNAIDS_gap_report

[38] Gorina E, Agadjanian V, Zotova N. Migrant women’s economic success in Russia: Objective reality and subjective assessment. Journal of Ethnic and Migration Studies. 2018;44(9):1584–603. 30245577 10.1080/1369183X.2017.1333410 PMC6147256

